# Oral supplementation with fish cartilage hydrolysate accelerates joint function recovery in rat model of traumatic knee osteoarthritis

**DOI:** 10.1002/fsn3.2244

**Published:** 2021-04-10

**Authors:** Yves Henrotin, Christophe Antoine, Elodie Zwerts, Thibaut Neutelings, Elodie Bouvret

**Affiliations:** ^1^ Artialis SA, Tour GIGA CHU Sart‐Tilman Liège Belgium; ^2^ Bone and Cartilage Research Unit Center for Interdisciplinary Research on Medicines, Arthropôle Liège Institute of Pathology University of Liège, CHU Sart‐Tilman Liège Belgium; ^3^ Physical Therapy and Rehabilitation Department Princess Paola Hospital, Vivalia Marche‐en‐Famenne Belgium; ^4^ Abyss Ingredients Caudan France

**Keywords:** cartilage, knee, osteoarthritis, pain

## Abstract

The objective of this study was to evaluate the effects of oral fish cartilage hydrolysate (FCH) on symptoms and joint tissue structure in rat developing osteoarthritis induced surgically. Osteoarthritis was induced in the right knee of mature male Lewis rats (*n* = 12/group) by surgical transection of the anterior cruciate ligament (ACLT) combined with partial medial meniscectomy (pMMx). Two weeks after surgery, rats were treated orally with either control (sterile H_2_O) or FCH for four weeks. Pain and function were assessed by dynamic weight‐bearing test (incapacitance test), electronic Von Frey (EVF; hindpaw allodynia threshold), and pressure algometer (knee allodynia threshold). Time and groups differences at each time point were evaluated using a mixed model. The histological features were evaluated eight weeks after surgery using OARSI score. Mann–Whitney test nonparametric test was applied to compare OARSI score. ACTL/pMMx surgery significantly reduced weight‐bearing and increased allodynia and sensitivity thresholds of the operated paw/knee. Globally, FCH improved these parameters faster, but no significant difference between control and FCH groups was observed. Eight weeks after surgery, rats developed moderate OA lesions. Compared with control, FCH did not significantly modify OA lesion severity assessed using the OARSI score. In this mechanically induced OA model, 4 weeks of supplementation with FCH had no significant effect on cartilage lesion, but tends to accelerate pain relief and joint function recovery. This positive trend may have opened the way for further investigation of FCH as potential treatment of joint discomfort associated with OA.

## Graphical abstract

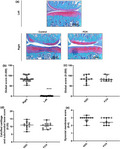

## 1 INTRODUCTION

Osteoarthritis (OA) is a common painful condition that imposes a substantial burden on individuals, healthcare systems and society. Its prevalence increases due to population aging and growing number of overweight people (Johnson & Hunter, 2014; Loeser et al., 2012). At this time, symptoms‐focused OA management requires an association of nonpharmacological and pharmacological modalities (i.e., analgesic drugs or intra‐articular injections of corticosteroid or hyaluronic acid (HA)) should be to relieve pain and to improve joint function and quality of life of patient with limb OA (2014; Hochberg et al., 2012; Jevsevar, 2013; McAlindon et al., 2014; Michael et al., 2010). Unfortunately, none of these treatments may be considered as a disease‐modifying drugs that are to say capable of improving symptoms and delaying the progression of joint structural changes. There is an urgent need to test new synthetic or natural molecules on both symptoms and structural changes associated with OA.

Good quality food supplements represent a complementary option thanks to their low risk‐benefit ratio (Castrogiovanni et al., 2016). Several food supplements have been studied in variable quality randomized clinical trials, and the overall analysis including all trials showed that some supplements (i.e., avocado/soybean unsaponifiable extract, *B. serrata* extract, pycnogenol, *C. longa* extract, and Passion fruit peel extract) provide moderate and clinically meaningful short‐term effects on pain and function in patients with hand, hip, or knee OA (Lim et al., 2019; Liu et al., 2018).

New food supplement may also represent a new opportunity to slow down or reverse OA progression. Many researchers have focused their attention on fish skeleton extracts as they are rich in glycosaminoglycans (GAG), proteoglycans (PGs), and collagen peptides. A recent study showed that PGs originated from shark cartilage that showed sequence similarity with aggrecan core protein and epiphycan, improved clinical signs and modulated the inflammatory status of the joint in a rat model in which OA was induced by monoiodoacetate (MIA) (Ajeeshkumar et al., 2019). These PGs may play a protective role by modulating factors involved in cartilage catabolism and inflammation. It has been showed that the expression of interleukin (IL)‐10, an anti‐inflammatory cytokine (Helmark et al., 2010), was upregulated while that of Tumor Necrosis Factor (TNF)‐α, IL‐1β, Matrix metalloprotease (MMP)‐13, nitric oxide synthase (NOS)‐2, and cyclooxygenase‐2, (COX‐2), all factors promoting cartilage catabolism and/or inflammation, was reduced. A more recent study showed that glucosamine and fish collagen peptides (FCP) had protective effects on cartilage in a rabbit model of OA induced by anterior cruciate ligament transection (ACLT)(Ohnishi et al., 2013). Another in vitro research demonstrated that fish collagen hydrolysate (FCH) induced and maintained chondrogenesis in equine adipose‐derived stromal cells similarly to TGF‐β1 (Ohnishi et al., 2013). In a guinea pig model of OA, oral administration of fish and porcine collagen hydrolysate was reported to reduce morphological destruction of cartilage (Helmark et al., 2010).

In contrast, a study focused on the effects of three different size of FCH on porcine cartilage explant metabolism showed that in the presence of interleukin‐1β and oncostatin‐M, small (<3 kDa) and medium (3–10 kDa) fractions had additive effect with interleukin‐1β and oncostatin‐M on cartilage degradation, whereas large size (>10 kDa) had no effect (Ajeeshkumar et al., 2019; Boonmaleerat et al., 2018). This means that according their processing, FCH may have no effect or deleterious effects on cartilage metabolism. Therefore, before their clinical use, their effects have to be investigated in in vitro and in vivo preclinical models.

In this preclinical study, we have tested a FCH on the knee pain and mobility of rat with OA induced experimentally by the section of the anterior cruciate ligament (ACLT) associated with a partial medial meniscectomy (pMMx). To our knowledge, it was the first time that this FCH was tested in animal developing mechanically induced OA.

## 2 METHODS

### 2.1 Experimental model and study design

Young adult male Lewis rats (*n* = 24) were obtained from of Charles River Laboratories (Ecully, France) at the age of 12 weeks old. Rats were housed by two in polysulfonate ventilated cage of 904 cm^2^ (Sealsafe® Plus GR900. Leicester, UK) in compliance with the European Directive 2010/63/EU. Rats have access to food and water ad libitum. Room temperature (between 20°C and 24°C), humidity (between 45% and 65%), and light cycles (light is on between 7:30 a.m. and 7:30 p.m.) were controlled. The study was conducted by Artialis SA (Liège, Belgium). The study protocol received the approval of the Ethical Committee of the “Centre d’Economie Rurale” (CER) Marloie (Registration Number: PS‐2019‐NOV‐006).

OA model was induced in the right knee of all rats by ACLT combined with pMMx (Appleton et al., 2007; Gerwin et al., 2010; Kamekura et al., 2005). Briefly, a medial arthrotomy was performed. The patella was luxated laterally. The medial meniscus and anterior meniscotibial ligament (aMTL) were identified. The aMTL ligament was transected, and the spontaneous posterior retraction of the medial meniscus was observed validating the complete transection of the aMTL. The anterior horn of the meniscus was grasped and the anterior third was carefully excised. Then the anterior cruciate ligament (ACL) was transected. An anterior drawer was induced by levering the tibial plateau anteriorly with a lever type instrument, thus confirming the complete transection of the ACL.

### 2.2 Surgery corresponded to week (W) 0

In order to ensure animal welfare, all rats received buprenorphine (0.01–0.05 mg/kg, subcutaneous) before anesthesia induction. A periarticular injection of bupivacaine 0.5% was performed before wound closure. As postsurgical follow‐up, each animal received as follows:

- Sterile physiological serum (500 µl subcutaneous) after surgery.
- Meloxicam (subcutaneous) on the surgery day and one day after surgery.
- Analgesic treatment (Buprenorphin) immediately after surgery and maintained during 72 hr.

Supplementation was administered daily to each animal by oral gavage from the second week (W2) to the sixth week (W6) after surgery. The animals were distributed into two experimental groups (*n* = 12/group): 1) negative control group received sterile H_2_O; 2) test item group received FCH (Cartidyss NG; 103.33mg/kg; Abyss Ingredients, France). FCH consists in a water‐soluble powder obtained from a standardized manufacturing process based on an enzymatic hydrolysis of marine fish cartilage without preservative nor processing aids. Its natural composition is unique, with collagen peptides (< 3 kDa), chondroitin sulfate and minerals, respectively, 65%, 26%, and 9%, given as an indicative value. A solution of FCH was prepared fresh each day of supplementation by solubilizing the powder in sterile H_2_O to obtain the desired concentration.

**FIGURE 1:**
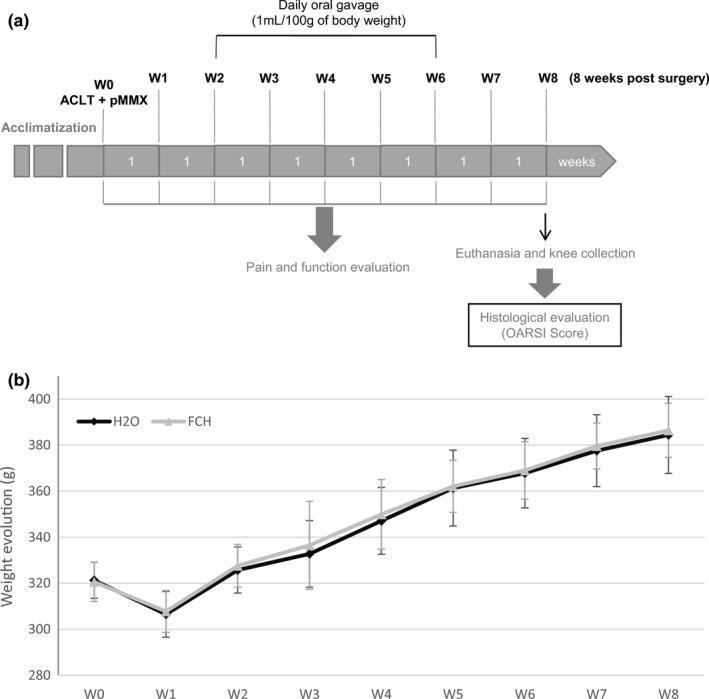
a. Schematic representation of the experimental design. b. Body weight evolution. Monitoring of the body weight (in g) of all animals all along the duration of the study (data are mean ± *SD*). 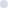 = FCH; ● = H_2_O

Test and reference items were administered daily between W2 and W6 by oral gavage using a feeding tube connected to 5 ml syringes filled with the appropriate volume according to the body weight (b.w.) of each animal (1ml/100g b.w.). Animals were followed during eight weeks after knee surgery (Figure 1a for schematic representation of the study design).

### 2.3 Pain and function assessment

Pain and function were assessed through three different measures. First, the weight bearing of animals was evaluated using the dynamic weight‐bearing test (DWB, Bioseb, France). This incapacitance test precisely measures changes in postural equilibrium in rodents by assessing their weight distribution on each one of their four paws by a sensor made of 2000 independent load cells of very high sensitivity. It gives a measure of the postural deficit when the animal is moving spontaneously in a transparent cage during a five‐minute experiment. The rear left/rear right paw (rear L/R) ratio weight bearing and the rear right paw (operated) weight bearing were extracted from the acquired data. Second, the mechanical allodynia threshold on the rear right (operated) and left paws was measured using the electronic von frey (EVF, Bioseb, France). The system consists in a plastic box (17 × 11 × 13 cm) placed on an elevated horizontal wire mesh stand. Animals were placed in the box and a stimulation of the central edge of both hind paws was performed with a portable force transducer fitted with a plastic tip. Paw withdrawal caused by the discomfort was registered as a response (maximal force applied in g). Third, the small animal algometer (SMALGO, Bioseb, France) was used to measure the threshold sensitivity of the knee as previously described (Sekar et al., 2020). This pressure‐based analgesimeter fits on the researcher‘s finger (thumb or index) and assesses the sensitivity threshold of the knee joint (in grams). The user applied progressive mechanical pressure on the knee joint with his own finger and increases the stimulation until he obtains a reaction from the animal (scream, shudder, paw removal...); then, the stimulation is stopped and the measure registered.

All animals were monitored on the three different measure systems once a week from W0 (before surgery, reference time point) to W8 (eight weeks after surgery and before euthanasia). W2 corresponded to the reference time point before supplementation administration.

### 2.4 Histological evaluation of knee cartilage and synovial membrane

Immediately after euthanasia, knee joints were collected and fixed in 4% paraformaldehyde (PFA). After appropriate fixation and decalcification (DC2 decalcifier, QPath), frontal histological sections of 5 µm were performed with a standard microtome on paraffin‐embedded samples. Samples were prepared from three sections 200 µm apart in weight‐bearing area and stained with Safranin‐O/Fast green.

The histological analysis of the four knee compartments (medial and lateral condyles and plateaus) was performed in accordance with the OARSI recommendations (Gerwin et al., 2010). The following parameters were evaluated as follows: cartilage matrix loss width (score 0–5 for superficial, intermediate and deep zones of cartilage; 3 values for each compartment); cartilage degeneration score (score 0–5 for outer third, middle third and inner third of cartilage; 3 values for each compartment); significant cartilage degradation (representing the loss of more than 50% of the tissue) width (score 0–5); total cartilage degeneration width (corresponding to any kind of cartilage modification) (score 0–5); calcified cartilage and subchondral bone score (0–5); osteophyte score (score 0–4); synovial membrane reaction (score 0–4). All widths were estimated as follows with a semiquantitative score: 0 = no modified tissue, 1 = modification concerning less than 10% of the total width; 2 = modification concerning 10%–25%; 3 = modification concerning 25%–50%; 4 = modification concerning 50%–75%; and 5 = modification expanding over 75% of the total width of the concerned tissue. Two trained experts, blinded for the supplementation groups, scored each slide under a light microscope. The histological global score obtained was the sum of all scores from the four compartments of the knee joint (total score 0–200).

Each compartment of the knee has been scored independently: medial and lateral femoral condyles and medial and lateral tibial plateaus. All scores were recorded and analyzed alone or in combination. The global score corresponded to the sum of the score of each criterion for the entire knee.

### 2.5 Statistical analysis

First, the distribution of the quantitative data was investigated. In case of non‐normality distribution, nonparametric tests were used.

DWB results were analyzed using SAS Version 9.4. Time evolution of the weight distribution between W0 and W8 in one hand and at the beginning of the supplementation (from W2 to W8) on the other hand was investigated and compared between the two groups. To this aim, a mixed model was fitted to the data to test. The covariates included in the model were the time (considered as a qualitative variable, W0 or W2 was considered as the reference) and an interaction term with the group indicator (controls group). This statistical method allowed the comparison of response curves between supplementations while accounting for repeated data within individuals. Time differences in both groups and group differences at each time point were evaluated with this statistical approach. Data are presented as mean and standard deviation. Considering the number of comparisons performed for each data set, the significance level was calculated at 0.001.

Histological data were analyzed with GraphPad Prism version 8. Normality of the data distribution was evaluated. When non‐normality was observed, nonparametric *t* test was applied to data that did not pass the normally test (Mann–Whitney test). Nonparametric data are presented as median and interquartile range (Q1–Q3) and individual points are presented on the graph. The significance level was considered below 0.05.

## 3 RESULTS

### 3.1 Weight evolution and animal behavior

The weight evolution of all animals was followed during the in‐life phase (Figure 1b). Before surgery, the mean weight was 321.3 ± 7.8 g in the control group and 320.6 ± 8.6 g in the treated group. Curves showed a non‐significant loss of body weight one week after surgery followed by a constant weight gain for all study groups until the end of the study.

All animals showed normal behavior, appearance or food and water intake after test or reference items oral administration. Based on the evaluated parameters, no signs of toxicity linked to the tested compound were observed in both groups.

### 3.2 Weight‐bearing assessment

**FIGURE 2:**
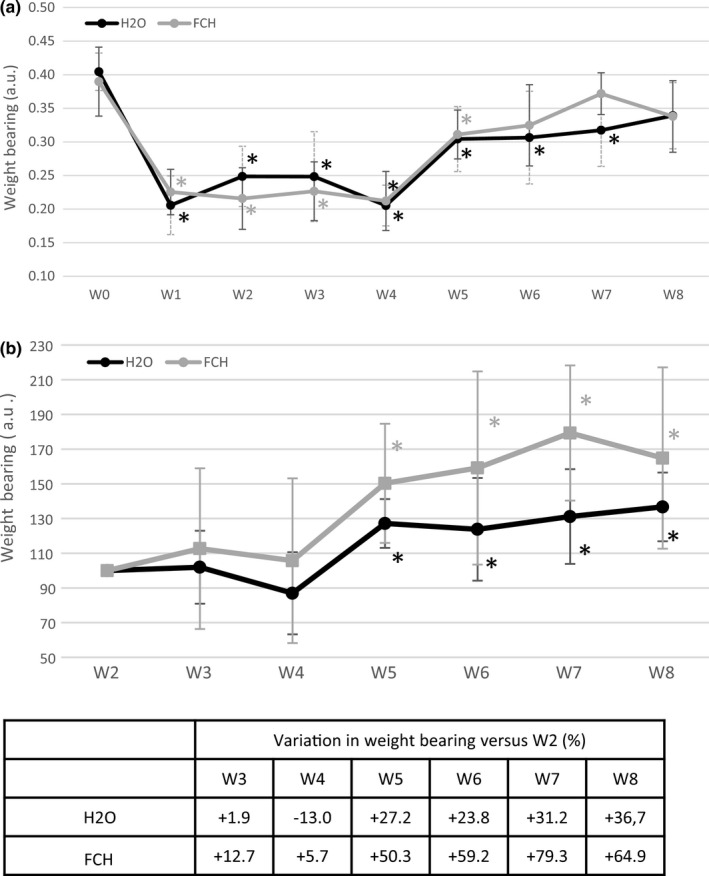
Evolution of rear right pain/joint function measured by dynamic weight‐bearing test. a. Evolution of rear right weight‐bearing over time in both groups (*n* = 12/group) from W0 (before surgery) to W8. Data were normalized by body weight. b. Evolution of rear right weight‐bearing over time in both groups (*n* = 12/group) versus supplement initiation at W2. Results are presented as mean ± *SD* at each time point (W corresponding to weeks after surgery) for each group (longitudinal analysis model for each group at each time point, *: p‐value ≤ 0.001 versus W0 was considered as significant). 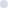 = FCH; ● = H_2_O

The weight bearing on the rear right (operated) paw (Figure 2a) that reflected the consequence of the ACLT/pMMx surgery was significantly decreased one week after surgery in both groups. The analysis was focused on the evolution of each parameter between the two study groups after supplement initiation (W2) and presented on Figure 2b. Weight bearing significantly increased at W5 in both group (+ 27.2% in H2O; + 50.3% in FCH group; *p* < .001) but to a higher magnitude in FCH group compared with H_2_O group since W5 (Figure 2b). The difference between groups tended to increase with time, suggesting that FCH‐treated rats recovered faster than controls. At final time point W8, controls displayed an increase of 37% of weight bearing of the operated limb while animals treated with FCH showed an increase of 65%. However, no significant difference between groups was recorded.

### 3.3 Rear paws mechanical allodynia threshold

**FIGURE 3:**
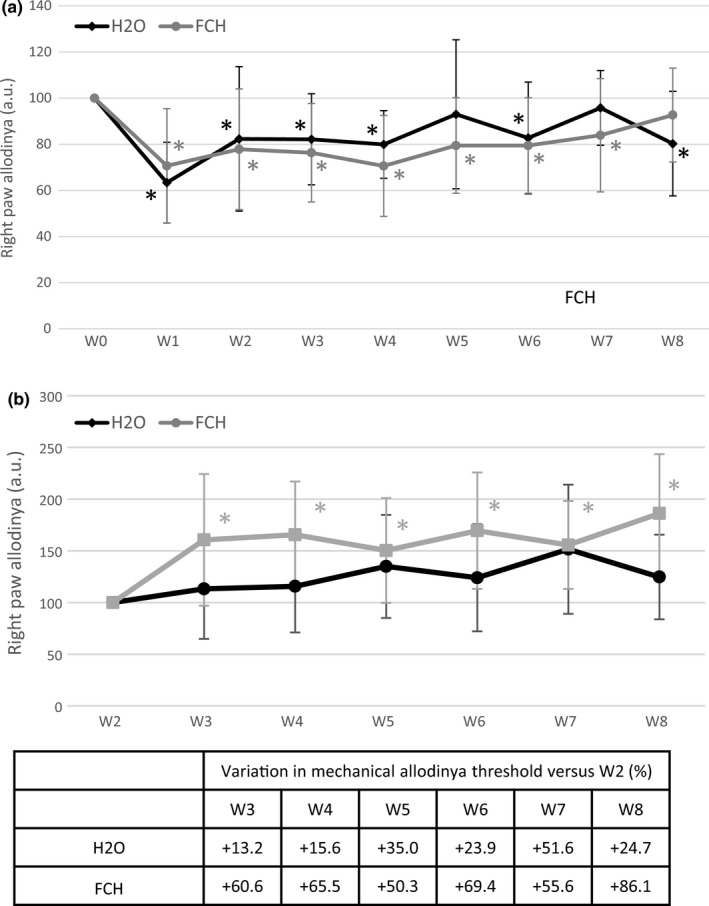
Rear right paw mechanical allodynia threshold evolution over time in both study groups. a. Evolution of rear right paw allodynia over time in both groups (*n* = 12/group) from W0 (before surgery) to W8. Data were normalized by body weight. b. Evolution of rear right weight sensitivity over time in both study groups (*n* = 12/group) versus supplement initiation at W2. Results are presented as mean ± *SD* at each time point (W corresponding to weeks after surgery) for each group (longitudinal analysis model for each group at each time point, *: p‐value ≤ 0.001 versus W0 was considered as significant). 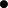 = FCH; ● = H_2_O

The mechanical allodynia threshold of the right (operated) and left paws was measured by EVF. The values from left paws remained quite constant over time meaning that neither the model nor the supplementation affected nonoperated limb mechanical sensitivity (data not shown). One week after surgery (W1), the mechanical allodynia threshold on the right paw decreased significantly in both groups (*p* < .001) (Figure 3a). The mechanical threshold was decreased by 1.7‐ and 1.5‐fold in control (H_2_O) and FCH, respectively.

When considering W2 as a reference (Figure 3b), right paw mechanical allodynia of controls did not show any significant variation over time. In contrast, mechanical sensitivity level of the right paw significantly increased in FCH group (*p* <.05), indicating that allodynia decreased in that group. No statistical difference between groups was observed.

### 3.4 Knee allodynia threshold

**FIGURE 4:**
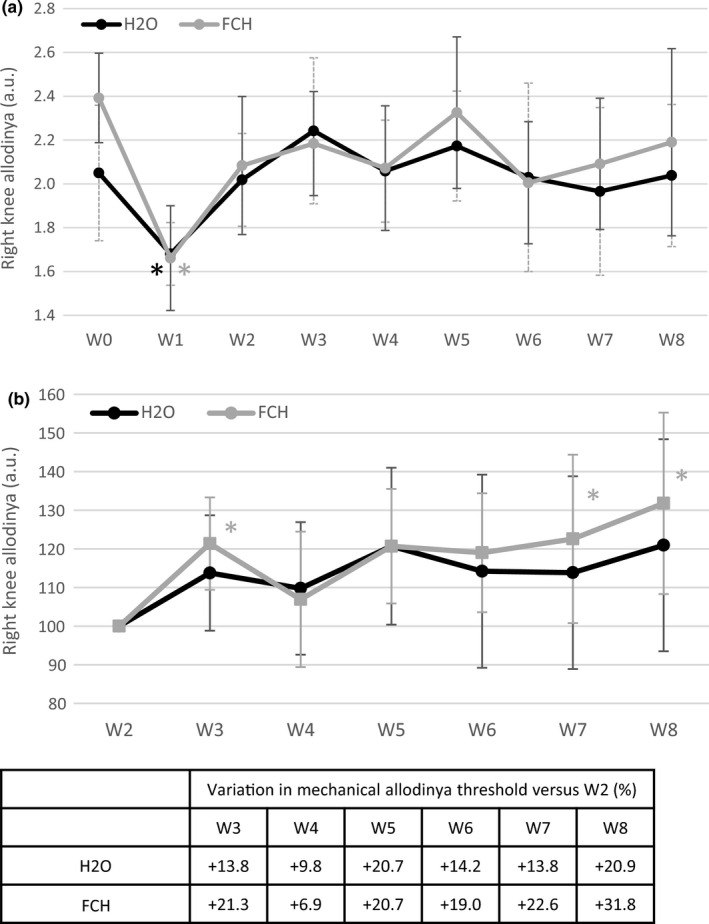
Right knee mechanical allodynia threshold evolution over time in both study groups. a. Evolution of rear right knee allodynia over time in both groups (*n* = 12/group) from W0 (before surgery) to W8. Data were normalized by body weight. b. Evolution of right knee mechanical allodynia threshold over time in both study groups (*n* = 12/group) versus supplement initiation at W2. Results are presented as mean ± *SD* at each time point (W corresponding to weeks after surgery) for each group (longitudinal analysis model for each group at each time point, *: p‐value ≤ 0.001 versus W0 was considered as significant). 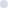 = FCH; ● = H_2_O

The pain threshold remained quite constant over time in the nonoperated (left) knee, meaning that neither the model nor the supplementation affected mechanical sensitivity of noninjured knee (data not shown). ACTL/pMMx surgery induced a significant decrease in the pain threshold in the operated (right) knee at W1 in both study groups: −143.4 g, *p* < .001; FCH: ‐ 215.3 g, *p* < .001) and progressively returned to normal level with time (Figure 4a).

When considering W2 (treatment starting) as reference (Figure 4b), a significant increase in the knee sensitivity threshold was observed in FCH group at W3 right after supplementation initiation and at W7 and W8 (*p* < .05). No such significant modification was observed in control group. No difference between groups was observed.

### 3.5 Histological analysis of the osteoarthritic knee joint

**FIGURE 5:**
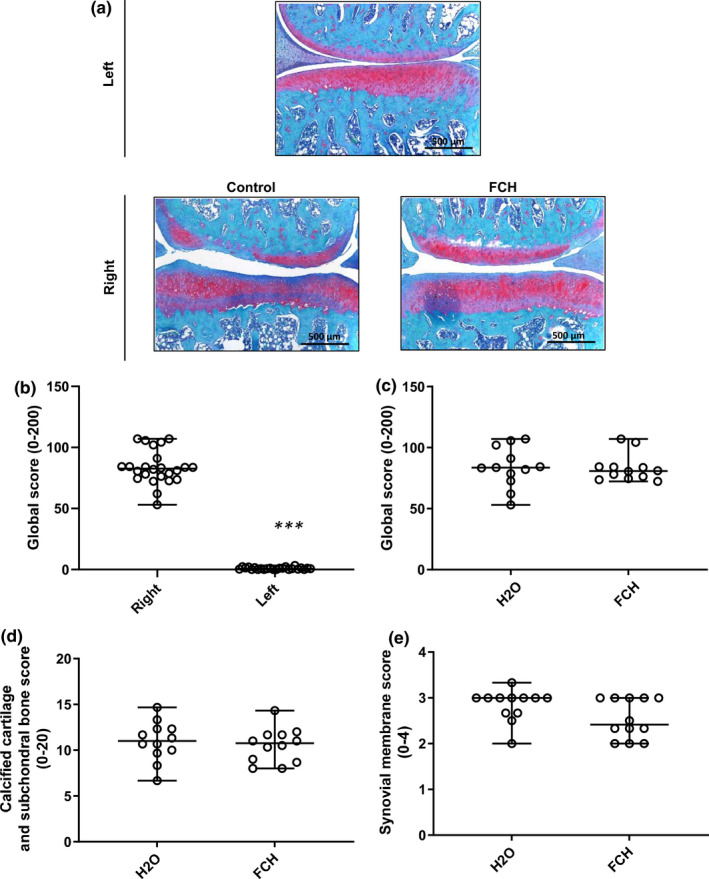
Histological quantification of the OA lesions: OARSI histological global score. a, Safranin O/Fast Green staining of representative knee section from the medial compartment. Left knee (upper panel) and right knee from H_2_O‐treated and FCH‐treated groups (lower panels) are presented (5‐µm thick section). b, OARSI score of the right and left knees for the H_2_O‐treated group. Data were compared using a Mann–Whitney U test for nonparametric values (***: *p* <.0001 was considered as significant). c, Global score of the right‐operated knee for both study groups. Each open dot represents the OARSI histological global score for one rat. d, OARSI score for calcified cartilage and subchondral bone. e. OARSI score for synovial membrane. Groups were compared using a Mann–Whitney U test for nonparametric values (*p* =.681, ns). Data are presented as median (horizontal bar) and interquartile range (Q1–Q3; vertical bars)

Safranin O/Fast Green staining of both knees of each animal was performed eight weeks after surgery (W8, Figure 5a). The OARSI global score dramatically decreased in the operated (right) knees (median (Q1–Q3): 83.50 (53.00–107.00) compared with the nonoperated (left) knee (median (Q1–Q3): 0.50 (0.00–3.33; *p* < .001) (Figure 5b). No significant difference between study groups was observed in the nonoperated (left) knee (data not shown). The OARSI global score revealed moderate OA lesions in both study groups (Figure 5c). However, no significant differences were reported between the study groups. The OARSI score for calcified cartilage and subchondral (Figure 5d) as well as the OARSI score for synovial membrane (Figure 5e) did not show any significant difference between study groups.

## 4 DISCUSSION

To our knowledge, this is the first study investigating oral fish cartilage hydrolysate FCH on symptoms of experimental rat OA model. The product tested was a skate fish cartilage hydrolysate containing collagen peptides and GAG. FCH was administered at a dosage mimicking administration in human (1 g/day). The aim of this study was to follow an administration scheme that would be as close as possible as real life in OA patients. In the experimental model used, OA was induced in the knee of rat by the section of the anterior cruciate ligament and partial medial meniscectomy. The ACLT/pMMX‐induced nociceptive behavior was characterized by a decrease in mechanical allodynia threshold and decrease weight‐bearing distribution in the injury hind paw. Therefore, an increase in this operated hind limb weight bearing can be interpreted as resulting from decreased pain and improved joint function. Further, 8 weeks after surgery, rat develops mild to moderate cartilage lesions characterized by cartilage surface fibrillation, loss of the extracellular matrix GAG, and thinning of cartilage. These nociceptive behaviors and structural alterations closely resemble those of human OA. Interestingly, weight bearing, mechanical allodynia, and knee pain threshold improved faster and more in FCH group than in control (H_2_O) group. Altogether, these data suggest that FCH decreases pain and improves animal mobility. This can be explained by the biological activities of FCH ingredients on inflammatory mediators, some of them being involved in the physiopathology of inflammatory‐related pain. Indeed, glucosamine, chondroitin sulfate, or collagen hydrolysate have demonstrated to decrease IL‐1, nitric oxide (NO), or prostaglandin E_2_ (PGE_2_) in the synovial fluid of rat with OA in similar models (Wang & Cai, 2018; Wen et al., 2010). However, the difference between control (H_2_O) and FCH groups failed to be significant, meaning that despite a trend in favor of FCH, we cannot conclude on an analgesic effect of this product. The absence of significant effect between groups can be partly explained by the particularities of the OA model used and the treatment duration. In this model, we studied the effects of the product in a short time after surgery. We cannot exclude that what we are studying is the analgesic effect of the product on postsurgical inflammatory pain and not on the mechanical pain associated with OA. The treatment duration was limited to one month while in clinical practice this kind of product shows a slow progressive effect reaching significance after 3 to 6 months. Moreover, the positive trend seems to increase with the duration of the treatment. Therefore, we can hypothesize that a longer treatment with FCH would achieve the significance. For these reasons, we have to be careful before concluding on the effect or absence of effect of FCH on OA symptoms.

This ACLT/pMMx rat model is also known to generate moderate OA according to the histological criteria of OARSI score. Eight weeks after the surgery, a putative decrease in OARSI degeneration scores in the operated paw can be interpreted as the result of cartilage degradation prevention. Unfortunately, in this study we have not observed significant difference between FCH and control (H_2_O) in any histological OA feature. ACLT/pMMx rat model can be considered as rapidly destructive osteoarthritis. Therefore, the tested product has to counteract the mechanical stress and to slow‐down cartilage degradation in the presence of deleterious mechanical stimuli. This could explain that few food supplements have demonstrated to be efficient on this model. Moreover, histological evaluation was performed only once 8 weeks after surgery. This time point might be too late to observe a positive effect on the structure that could be correlated with the effect on pain and function.

## 5 CONCLUSION

In conclusion, oral skate fish cartilage hydrolysate tends to attenuate postsurgically nociceptive behavior such as mechanical allodynia and weight‐bearing distribution, in an experimental rat OA model. These findings may have opened the way for further investigations of skate fish cartilage hydrolysate as a potential solution to reduce joint discomfort associated with OA.

## ETHICAL STATEMENT

The study protocol received the approval of the Ethical Committee of the “Centre d’Economie Rurale” (CER) Marloie (Registration Number: PS‐2019‐NOV‐006). YH was the chairman of the board of Artialis SA and received consulting fees from Artialis SA, Tilman, Naturex, Nestlé, Seikagaku, Stemmatters, and Genequine. CA, EZ, and TN were employees of Artialis SA. EB was an employee of Abyss ingredients.
